# Remarkable Recovery After Delayed High‐Dose Methylprednisolone in a Rare Case of Penetrating Spinal Cord Injury

**DOI:** 10.1002/acn3.70405

**Published:** 2026-04-21

**Authors:** Honghong Wang, Shuang Liu, Jiale Wang, Kaiyang Xue, Shenwei Li, Zhongheng Du

**Affiliations:** ^1^ Department of Rehabilitation Medicine The People's Hospital of YuHuan Taizhou, Zhejiang China; ^2^ Department of Acupuncture The First Affiliated Hospital of Wenzhou Medical University Wenzhou, Zhejiang China

**Keywords:** case report, delayed treatment, methylprednisolone, neurological recovery, penetrating spinal cord injury

## Abstract

Traumatic spinal cord injury (TSCI) caused by sharp‐force penetration is exceptionally rare, and the use of high‐dose methylprednisolone (MP) remains highly controversial, especially beyond the conventional 8‐h treatment window. This case report describes a 30‐year‐old male with acute incomplete TSCI following a knife stab wound to the right neck. Despite no initial improvement, high‐dose MP (1000 mg/day for 3 days, tapered thereafter) was initiated 40 h post‐injury, along with mannitol. Remarkable neurological recovery was observed, including independent ambulation by post‐injury day 25. Follow‐up MRI showed significant hematoma absorption. This case suggests that delayed high‐dose MP may benefit selected TSCI patients, particularly those with penetrating mechanisms and ongoing secondary injury. It highlights the potential for extending the therapeutic window in specific scenarios and supports the need for further research into individualized treatment strategies for TSCI.

**Abbreviations**
MP: methylprednisolone
MRI: magnetic resonance imaging
TSCI: traumatic spinal cord injury

## 1 Introduction

Traumatic spinal cord injury (TSCI) is a catastrophic neurologic condition frequently resulting in long‐term sensorimotor and autonomic deficits [1]. Based on the etiology, it can be classified as traumatic or nontraumatic [2]. Research in 2016 showed that the incidence of Traumatic SCI is approximately 0.013% [3] and results in high medical costs [4]. Penetrating TSCI accounts for < 1% of all TSCI cases, with sharp‐force injuries being exceptionally rare (only 14 PubMed‐indexed reports to date). The use of high‐dose steroids in TSCI remains highly controversial. Current clinical guidelines recommend the early administration of high‐dose methylprednisolone (MP) within the 8‐h golden window following injury. Beyond this timeframe, the neuroprotective benefits are significantly diminished, while the risk of complications increases substantially [5]. Due to the inherent rarity of penetrating TSCI, there is a significant lack of robust clinical studies evaluating the efficacy and optimal timing of high‐dose MP in this specific context. Now we report on a 30‐year‐old male who sustained a knife stab wound to the right neck, developed acute incomplete TSCI, and was treated with high‐dose MP 40 h post‐injury. The patient's condition improved rapidly following the treatment. This case suggests that high‐dose MP may have a potential therapeutic effect on TSCI caused by sharp instrument injury.

## 2 Case Report

**FIGURE 1:**
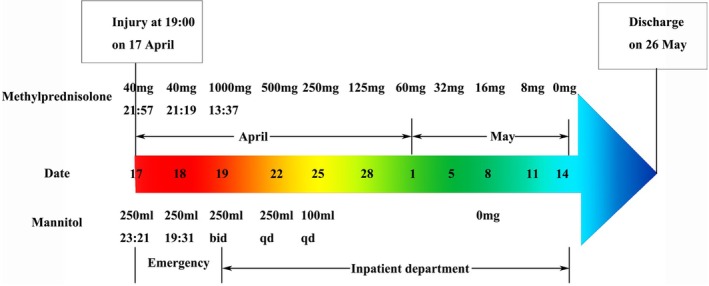
Patient injury and cervical spine magnetic resonance imaging. (A) A sharp wound approximately 3 cm in length on the right side of the patient's neck (indicated by the black arrow). (B) Magnetic resonance imaging from April 18, demonstrating a right‐sided spinal cord injury at the C7/T1 level and a corresponding hematoma within the spinal canal (highlighted by the red arrow). (C) Follow‐up imaging from May 5, showing resolution of the previously noted hematoma, with residual spinal cord injury at the C7/T1 level (indicated by the red arrow).

**FIGURE 2:**
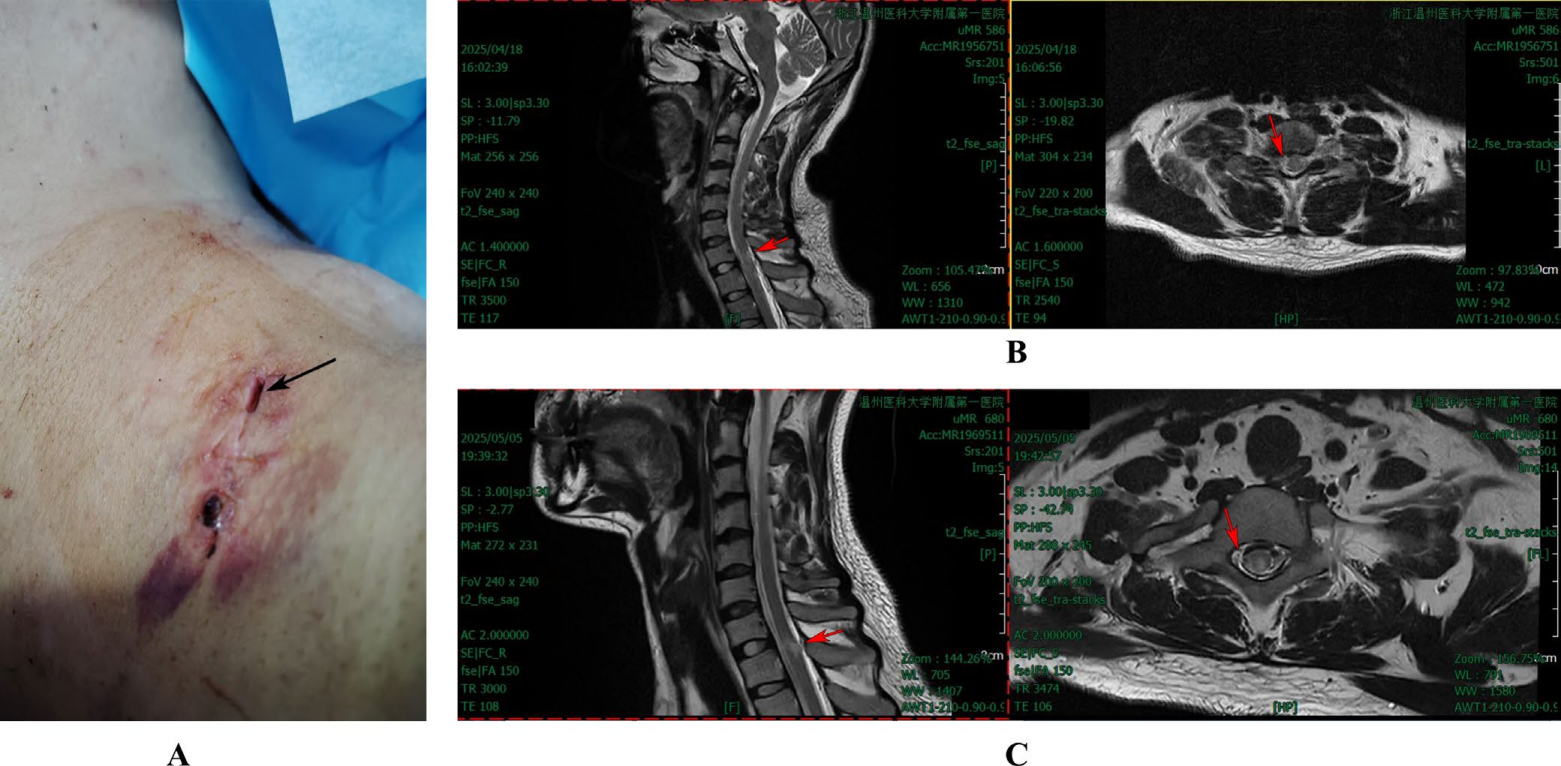
Patient Treatment Timeline and Usage of Methylprednisolone and Mannitol.

**FIGURE 3:**
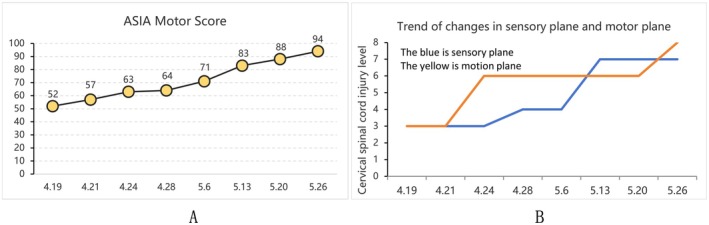
Rehabilitation score. (A) Represents the trend change of the total muscle strength scores of all key muscles in the ASIA assessment scale. (B) Represents the recovery of sensory and motor planes in the cervical segment.

A previously healthy 30‐year‐old male was stabbed in the right neck (Figure 1A) at 19:00 on 17 April 2025. Neurological examination on admission (21:57) revealed muscle strength of grade 2 in the right upper limb, grade 0 in the right lower limb, and grade 4 in both left upper and lower limbs, accompanied by diminished sensation in all four extremities and urinary retention. Initial management consisted of a 40 mg intravenous drip of MP followed by 250 mL of mannitol. Cervical MRI (18 April) demonstrated a right‐sided intramedullary lesion at C7/T1 without transection (Figure 1B). Conservative treatment was recommended by spine surgeons. Over the next 24 h, no clinical improvement was observed. At 40 h post‐injury (19 April, 11:00), the patient was transferred to our department. The patient's neurological status had improved modestly: muscle strength was graded 4 in the left limbs, 3 in the right upper limb, and 0 in the right lower limb; sensation was decreased below C3 on the right side and absent below C8; and urinary retention was present. After fully informing the patients and their families of the risks and benefits of using high‐dose MP, the patients strongly requested to accept the plan, so the treatment plan was formulated. A high‐dose MP protocol was initiated: 1000 mg administered via intravenous infusion daily for 3 days. Subsequently, the dose was tapered by half every 3 days (i.e., 500 mg daily for 3 days, followed by 250 mg daily for 3 days). Mannitol was administered concurrently for osmotic dehydration. (Figure 2) [6]. A comprehensive daily rehabilitation regimen was administered, integrating acupuncture, medium‐frequency electrical nerve stimulation, and exercise therapy. The acupuncture protocol targeted the paravertebral line (1.5 cun lateral to the posterior midline) from C6 to T2 as the primary points. Supplementary distal points included bilateral Quchi (LI11), Shousanli (LI10), Waiguan (TE5), Hegu (LI4) on the upper limbs, as well as Liangqiu (ST34), Xuehai (SP10), Zusanli (ST36), Xuanzhong (GB39), and Taichong (LR3) on the lower limbs. MRI of the cervical spine was reviewed on May 5, indicating that the hematoma had been significantly absorbed (Figure 1C). Serial clinical assessments showed progressive improvement (Figure 3). The patient achieved independent ambulation for the first time on 12 May following the injury (see Video S1). At the time of discharge (26 May), muscle strength had improved significantly (Figure 3A): grade 5 in the left limbs, grade 5 proximally and grade 3 distally in the right upper limb, and grade 4 in flexors and grade 5 in extensors of the right lower limb. Significant improvement in motor and sensory planes (Figure 3B). The patient can walk independently and has mild dependency in activities of daily living (see Video S1).

## 3 Discussion

This case suggests that delayed administration of high‐dose MP (at 40 h post‐injury) may yield a positive therapeutic effect in patients with traumatic spinal cord injury caused by sharp‐force penetration. The management of TSCI with high‐dose MP remains one of the most contentious topics in neurotrauma. While the National Acute Spinal Cord Injury Studies (NASCIS) trials suggested a potential benefit within an 8‐h window [7, 8], subsequent analyses and modern guidelines have strongly discouraged its routine use due to significant complications and lack of reproducible efficacy [9, 10]. However, our case presents a unique scenario: a patient with a penetrating injury who received MP markedly outside the conventional therapeutic window yet exhibited rapid neurological improvement. The rationale for the efficacy of delayed MP likely stems from the distinct pathophysiological mechanisms of penetrating versus blunt trauma. Unlike blunt TSCI, which involves diffuse concussion and rapid ischemic cascades that strictly limit the therapeutic window, penetrating injuries often result in focal anatomical disruption bordered by viable tissue, where secondary injury is driven by localized hematoma. Based on this, we hypothesize that the persistent vasogenic edema and ongoing inflammatory cascade observed on the Day‐2 MRI indicated an active secondary injury phase, potentially extending the window for effective anti‐inflammatory intervention. The delayed MP administration in this context may have mitigated this secondary damage, leading to the observed clinical recovery. Due to the disruption of the blood‐spinal cord barrier, inflammatory cells (such as macrophages, microglial cells, T cells, and neutrophils) infiltrate the injury site. These cells trigger the release of inflammatory cytokines (such as tumor necrosis factor [TNF] α, interleukin [IL]‐1α, IL‐1β, and IL‐6), whose levels peak at 6 to 12 h after injury and remain elevated for 4 days post‐injury [11]. In our patient, persistent edema on day‐2 MRI and lack of early recovery supported ongoing secondary injury. Mannitol, by reducing vasogenic edema and enhancing hematoma resorption, may synergistically enhance steroid efficacy. No infectious complications, gastrointestinal bleeding, or hyperglycemic crises were encountered during follow‐up. Nevertheless, vigilant monitoring remains mandatory given prior reports of steroid‐related morbidity.

This report has several limitations. First, it is a single case report, which inherently limits the generalizability of the findings. While the patient's remarkable neurological improvement was observed following the steroid administration, the influence of natural recovery processes cannot be entirely ruled out. Furthermore, the potential for survivorship bias must be acknowledged, as only cases with positive outcomes are more likely to be reported. Although the patient achieved independent ambulation by the first follow‐up at 26 days post‐discharge (see Video S1) and achieved functional independence in activities of daily living at the 102‐day follow‐up (September 4, see Video S1), the lack of more extended long‐term functional and imaging follow‐up remains a significant constraint. This prevents a comprehensive assessment of the sustained efficacy and potential late complications of the treatment regimen. In conclusion, this singular case demonstrates that high‐dose MP, even when initiated beyond the conventional 8‐h therapeutic window, may represent a potential therapeutic option for a select subset of TSCI patients, particularly those with penetrating injuries and ongoing secondary inflammation. It underscores the importance of individualized assessment and management in clinical decision‐making, moving beyond a rigid one‐size‐fits‐all approach. Ultimately, this report provides a rationale and direction for future research into delayed interventions targeting the secondary injury cascade in TSCI.

## Author Contributions

**Honghong Wang:** conceptualization, data curation, formal analysis, investigation, methodology, writing – original draft, writing – review and editing. **Shuang Liu:** data curation, investigation, resources, validation. **Jiale Wang:** investigation, resources, visualization. **Kaiyang Xue:** investigation, resources, software. **Shenwei Li:** supervision, validation, project administration. **Zhongheng Du:** conceptualization, funding acquisition, methodology, project administration, resources, supervision, validation, writing – review and editing.

## Funding

The authors have nothing to report.

## Ethics Statement

Written informed consent was obtained from the patient for publication of this case report.

## Consent

Written informed consent was obtained from the patient for publication of this case report and any accompanying images.

## Conflicts of Interest

The authors declare no conflicts of interest.

## Supporting information


**Video S1:** This video shows the patient's functional recovery at four time points. On May 12, he performs bedside standing and transfer with hand support and assisted wall walking, demonstrating severe lower limb weakness and balance impairment. On May 26, he walks without wall support but with a widened base and unsteady gait. On June 21, he walks independently with mild foot drop and slight asymmetry. On September 4, he achieves independent community ambulation with normalized gait, stable balance, and recovered fine motor skills including finger opposition, fist clenching, and ball grasping.

## Data Availability

The original contributions presented in this study are included in the article/Video S1. Further inquiries can be directed to the corresponding author(s).
